# Affective Stimuli for an Auditory P300 Brain-Computer Interface

**DOI:** 10.3389/fnins.2017.00522

**Published:** 2017-09-21

**Authors:** Akinari Onishi, Kouji Takano, Toshihiro Kawase, Hiroki Ora, Kenji Kansaku

**Affiliations:** ^1^Systems Neuroscience Section, Department of Rehabilitation for Brain Function, Research Institute of National Rehabilitation Center for Persons with Disabilities Tokorozawa, Japan; ^2^Center for Frontier Medical Engineering, Chiba University Inage, Japan; ^3^Biointerfaces Unit, Institute of Innovative Research, Tokyo Institute of Technology Yokohama, Japan; ^4^Brain Science Inspired Life Support Research Center, The University of Electro-Communications Chofu, Japan

**Keywords:** BCI, BMI, P300, EEG, affective stimulus

## Abstract

Gaze-independent brain computer interfaces (BCIs) are a potential communication tool for persons with paralysis. This study applies affective auditory stimuli to investigate their effects using a P300 BCI. Fifteen able-bodied participants operated the P300 BCI, with positive and negative affective sounds (PA: a meowing cat sound, NA: a screaming cat sound). Permuted stimuli of the positive and negative affective sounds (permuted-PA, permuted-NA) were also used for comparison. Electroencephalography data was collected, and offline classification accuracies were compared. We used a visual analog scale (VAS) to measure positive and negative affective feelings in the participants. The mean classification accuracies were 84.7% for PA and 67.3% for permuted-PA, while the VAS scores were 58.5 for PA and −12.1 for permuted-PA. The positive affective stimulus showed significantly higher accuracy and VAS scores than the negative affective stimulus. In contrast, mean classification accuracies were 77.3% for NA and 76.0% for permuted-NA, while the VAS scores were −50.0 for NA and −39.2 for permuted NA, which are not significantly different. We determined that a positive affective stimulus with accompanying positive affective feelings significantly improved BCI accuracy. Additionally, an ALS patient achieved 90% online classification accuracy. These results suggest that affective stimuli may be useful for preparing a practical auditory BCI system for patients with disabilities.

## Introduction

The brain-computer interface (BCI), also referred to as the brain-machine interface (BMI), translates brain signals into control signals for computers or machines (Wolpaw et al., 2002). Because the BCI is independent of muscle activity, it enables individuals with disabilities to control assistive devices or communicate with others. Brain signals acquired from invasive or non-invasive measurements have been used in the BCI research (Pfurtscheller et al., 2008). The primary approach for a non-invasive BCI is electroencephalography (EEG), where neurophysiological signals are recorded by an array of scalp electrodes.

In EEG-based non-invasive BCIs, sensory evoked signals that can be modulated by intention have been used. A popular system is the visual P300 BCI (Farwell and Donchin, 1988). This BCI provides both frequent and infrequent stimuli, and detects the EEG responses to the infrequent stimuli. These responses are called event-related potential (ERP), and contain a positive peak ~300 ms after the stimulus occurs, i.e., the P300. The visual P300 BCI has been used widely (Piccione et al., 2006; Salvaris and Sepulveda, 2009; Townsend et al., 2010). The visual P300 BCI has been proven applicable for persons with various types of paralysis, such as amyotrophic lateral sclerosis (ALS) (Nijboer et al., 2008; Ikegami et al., 2014).

For persons who have difficulty to control the gaze-dependent BCIs, various gaze-independent BCI techniques have also been proposed. For example, a gaze-independent visual speller (Blankertz et al., 2011), a gaze-independent steady-state visual-evoked-potential (SSVEP) based BCI (Lesenfants et al., 2014), a motor imagery based BCI (Pfurtscheller et al., 2006), a tactile P300 BCI (Brouwer and Van Erp, 2010), and an auditory P300 BCI (Halder et al., 2010; Höhne et al., 2011) have all been proposed. These options may be helpful for individuals with various disabilities and symptoms.

Among the gaze-independent BCIs, the auditory P300 BCI applications were proposed and evaluated in clinical studies. Sellers and Donchin employed word-based stimuli (“Yes,” “No,” “Pass,” and “End”) presented in visual and auditory modalities (Sellers and Donchin, 2006). The analysis showed that the offline classification accuracies of two out of three ALS patients were as high as those of able-bodied participants. Lulé et al. (2013) also evaluated the auditory P300 BCI with word-based stimuli (“yes,” “no,” “stop,” and “go”). Thirteen healthy subjects and one locked-in patient were able to communicate using their system. An auditory P300 speller using acoustically presented numbers was proposed (Furdea et al., 2009) and clinically evaluated for ALS patients online (Kübler et al., 2009).

Several techniques and stimulus types have been proposed to improve the auditory BCI accuracy. Klobassa et al. (2009) employed environmental sounds (bell, bass, ring, thud, chord, and buzz). Schreuder et al. (2010) developed a BCI that provided spatial auditory stimuli from five speakers located around a participant, providing better classification accuracy than the single-speaker condition. They also proposed the Auditory Multi-class Spatial ERP paradigm that enabled users to spell letters by taking advantage of spatial cues (Schreuder et al., 2011). Hill and Schölkopf (2012) proposed streaming stimuli that were intended to elicit ERP and auditory steady-state response. Höhne et al. (2012) found that “natural” auditory stimuli, such as short, spoken syllables, improved the classification accuracy of the BCI. Simon et al. (2015) proposed a BCI that used spatial auditory cues with animal voices.

Affective stimuli may be effective at improving the performance of EEG-based auditory P300 BCIs. Although affective auditory stimuli have not been applied to auditory P300 BCIs before, affective facial images were used as stimuli for a visual P300 BCI, and did improve classification accuracy (Zhao et al., 2011). Additionally, affective visual stimuli have been shown to enhance the P300 (Polich and Kok, 1995; Delplanque et al., 2004). A musical emotion study revealed that a violation of harmony increased emotion and enhanced the late component of the P300 (Steinbeis et al., 2006). Based on these studies, we hypothesized that affective auditory stimuli should also improve classification accuracy.

In this research, we used positive and negative affective sounds (PA: a meowing cat sound, NA: a screaming cat sound) as stimuli for an auditory P300 BCI. We expected that the two affective stimuli would modulate ERPs and improve the classification accuracy of the BCI. Permuted stimuli of the positive and negative affective sounds (permuted-PA, permuted-NA) were also used for comparison by keeping the features hidden in affective sounds, except those in the time series. We used a visual analog scale (VAS) to measure positive and negative affective feelings in the participants. Offline analysis was applied to investigate the effects of affective stimuli in detail. We also conducted an online experiment with an ALS patient to validate the methods proposed in this study.

## Materials and methods

This study was approved by the institutional ethics committee at the National Rehabilitation Center for Persons with Disabilities, and all participants provided written informed consent according to institutional guidelines. All experiments were performed in accordance with the approved guidelines.

### Experiment 1: effect of affective stimuli with able-bodied participants

#### Participants

Fifteen participants (aged 29 ± 7.2 y.o.; 7 women) took part in this experiment. Fourteen participants were right-handed and one participant was ambidextrous, according to the Japanese version of the Edinburgh Handedness Inventory (Oldfield, 1971).

#### Experimental design

**Figure 1:**
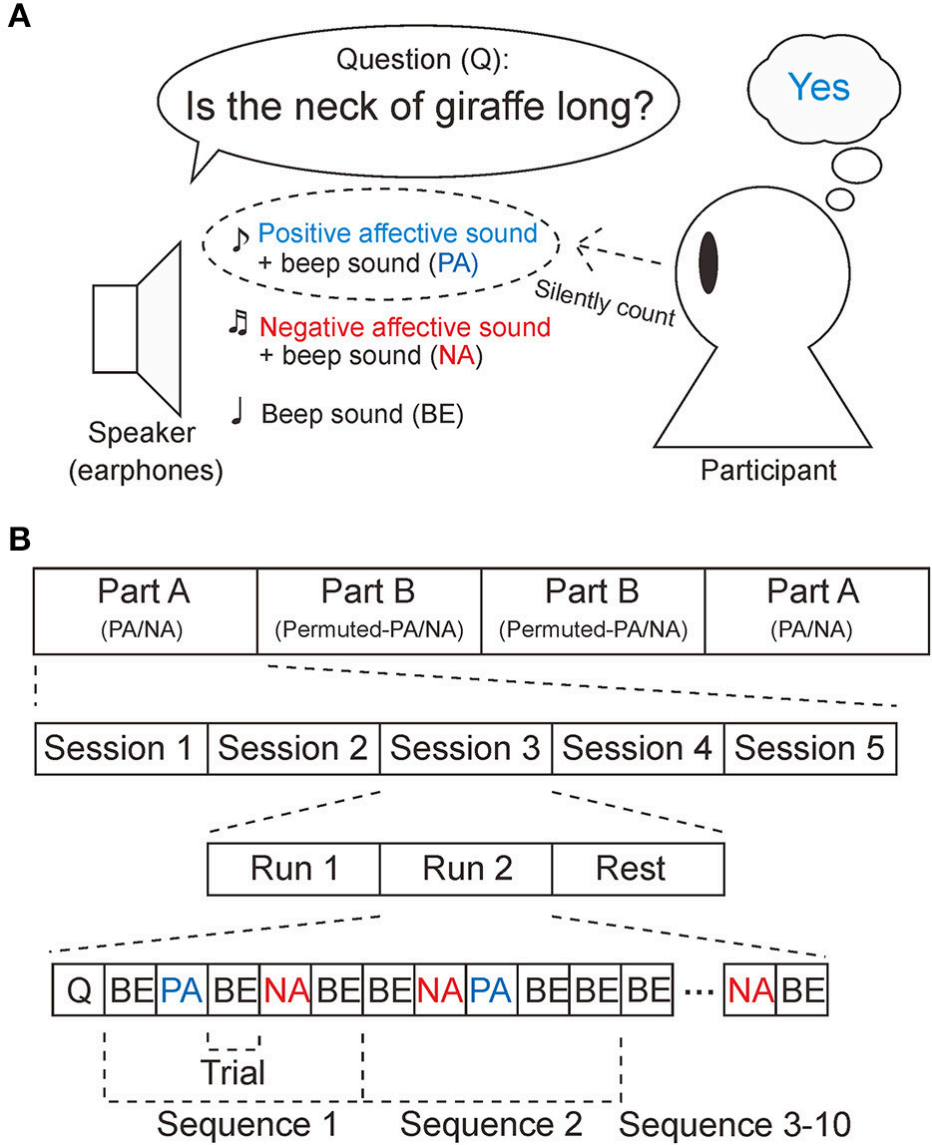
Task and experimental design. **(A)** Participants were asked to perform the oddball task in response to the sounds played through the earphones. First, a question to be answered “yes” or “no” was provided. If the answer was “yes,” the participants counted the positive affective sounds (PA). If the answer was “no,” the participants counted the negative affective sounds (NA). Note that the PA stimuli and NA stimuli were mixed with a 1,000 Hz sinusoidal waveform (beep sound). In the control experiment, PA and NA were replaced by permuted-PA and permuted-NA, respectively. **(B)** This experiment consisted of four parts (part A × 2, part B × 2), five sessions, and two runs. Part B was designed as a control experiment. In part A, PA, NA, and beep sounds (BE) were provided, while permuted-PA, permuted-NA, and B were provided in part B. Participants were asked to rest between sessions, At the beginning of a run, a yes/no question (Q) was provided. EEG measurements were taken during the tasks for PA, NA, permuted-PA, and permuted-NA.

The P300 BCI system used provides auditory stimuli. Participants were required to perform an oddball task, as shown in Figure 1A (Farwell and Donchin, 1988; Polich, 2007). First, the BCI system provides a yes/no question from earphones, such as “Is the neck of a giraffe long?” Next, three auditory stimuli, a positive affective sound attributed to “yes,” a negative affective sound associated with “no,” and a 1,000-Hz sinusoidal waveform, were presented pseudo-randomly. The sound that was the correct answer to the question was the target sound. Participants were asked to count the target sound 10 times in total, while ignoring the non-target sounds. The duration of the sounds was 500 ms and the stimulus onset asynchrony (SOA) was 1,000 ms. Because the SOA was longer than the duration of the sounds, the effects of ERP overlap could be reduced. The experiment was designed to be conducted within 3 h, in order to avoid fatigue.

Positive affective (PA) and negative affective (NA) sounds were associated with the answers “yes” and “no,” respectively. A meowing cat sound was used as the PA sound, while a screaming cat sound was used as the NA sound. The meowing cat sound is available at the Sound Effect Lab (cat-cry2.mp3 on http://soundeffect-lab.info/), and the screaming cat sound is available at SoundBible.com (Angry Cat-SoundBible.com-1050364296.wav on http://soundbible.com/). These stimuli were manually trimmed to 500 ms and sound pressure levels were equalized. In addition to the PA and NA sounds, a 1,000-Hz sinusoidal waveform was prepared as a beep sound. The stimuli were sampled at 44,100 Hz. The sinusoidal waveform (1,000 Hz) was mixed with the stimuli. The sinusoidal waves were rounded with a 50-ms linear slope at the beginning and end of the sounds.

Permuted stimuli of the positive and negative affective (permuted-PA and permuted-NA) sounds were also prepared for comparison. Time-domain scrambling (http://www.ee.columbia.edu/ln/rosa/matlab/scramble/) was used for the permuted stimuli. The sound was chopped into a 50%-overlap short window set, then Hanning-windowed and shuffled. The window length for scrambling was 10 ms and the radius of window shuffling was set to 250 ms. The permuted sounds were mixed with the non-permuted sinusoidal waveform.

As shown in Figure 1B, the experiment consisted of four parts (part A × 2, part B × 2). In part A, PA and NA sounds were provided as positive and negative stimuli. In part B, the permuted-PA and permuted-NA sounds were used instead. The experiment was conducted in the order ABBA or BAAB, pseudo-randomly. Prior to starting each part, the positive, negative, and beep sounds were played once. Then, the positive (PA in part A, or permuted-PA in part B) and negative (NA in part A or permuted-NA in part B) stimuli were associated with the answers yes and no. Each part consisted of five sessions. In each session, participants were required to perform an oddball task for two runs. At the beginning of the run, a question to be answered yes or no was played from the earphones. The participants were asked to rest for a few minutes between sessions. In each part, questions to be answered yes or no were provided five times (10 times in total), respectively. The sequence of the stimuli was repeated 10 times. No feedback was provided during the experiment.

#### EEG recording

The BCI system consisted of a laptop computer, a digital sound interface, earphones, a display, and an EEG amplifier. The instructions and BCI stimuli were presented through earphones (Etymotic ER4 microPro; Etymotic Research, Elk Grove Village, IL). The sounds were processed with an external sound card (RME Fireface UC; Audio AG, Haimhausen, Germany). All stimuli were controlled via the laptop computer using MATLAB/Simulink (Mathworks Inc., Novi, MI) and the Psychophysics Toolbox. Using g.USBamp (Guger Technologies, Graz, Austria), EEG signals were recorded from C3, Cz, C4, P3, Pz, P4, O1, and O2, according to the “10–20” system. The sampling rate for the EEG recording was 128 Hz. All channels were referenced to the left mastoid and grounded to the right mastoid. These electrode locations are based on past P300 EEG studies (Comerchero and Polich, 1999; Takano et al., 2009; Chang et al., 2013). A hardware bandpass filter (0.1–60 Hz) and notch filter (50 Hz) were applied. Non-adhesive solid-gel electrodes were used (Toyama et al., 2012). Fixation of eye gaze and images for the task instructions were presented via a display (Acer XB270H, Acer Inc., New Taipei City, Taiwan).

#### EEG analyses

Offline classification accuracies for positive and negative stimuli were computed using 10-fold cross-validation. ERPs were obtained when auditory stimuli were presented. This data was processed separately in target and non-target trials. When positive affective stimuli were used, a binary classifier was trained on the positive target (PA sounds) trials and non-target (NA and beep sounds) trials. In this case, 9 sessions of data were used for training while the rest was used for test data in the cross-validation. Thus, a classifier was trained on 90 target ERPs and 360 non-target ERPs. The test data containing positive target trials and non-target trials was then classified. Similarly, when negative affective stimuli were used, a binary classifier was trained on the negative target (NA sounds) trials and non-target (PA and beep sounds) trials, and then the test data containing negative target trials and non-target trials was classified. This analysis was applied to the data obtained from parts A and B. In this manner, the classification accuracies of PA, NA, permuted-PA, and permuted-NA were calculated.

In the offline classification, 700 ms epochs of EEG were extracted, smoothed (4 sample points), bandpass-filtered (Butterworth, 0.1–25 Hz), downsampled to 25 Hz, and vectorized. Stepwise linear discriminant analysis (SWLDA; *p*_in_ = 0.1, *p*_out_ = 0.15) was then used (Krusienski et al., 2008). The classifier was used to calculate the outputs corresponding to positive and negative stimuli (1: positive, 2: negative). Given the weight vector of SWLDA, ***w***, the outputs for positive and negative stimuli were computed as:

(1) $$\hat{i}={\text{argmax}}_{i}\sum_{r=1}^{10}w\;\cdot \;x_{r,i},$$

where ***x***_*r, i*_ is the preprocessed data of the *r*^th^ intensification sequence of the *i*^th^ stimulus (*i* ∈ {1, 2}). Thus, the classifier was trained on 90 target ERPs (9 sessions × 1 trial × 10 sequences) and 360 non-target ERPs (9 sessions × 4 trials × 10 sequences).

We also analyzed the offline classification accuracy for each part, meaning the accuracy for part A (PA + NA) and part B (permuted-PA + permuted-NA). In this case, the SWLDA classifier was trained on target ERPs and non-target ERPs without discriminating between PA and NA (also permuted-PA and permuted-NA). The parameters used in this analysis were the same as those used in the offline analysis for PA, permuted-PA, NA, and permuted-NA.

For data visualization, averaged waveforms were preprocessed in the same manner, except for changes in artifact removal and downsampling. Waveforms with artifacts exceeding ±100 μV were removed. In order to analyze the ERPs, the waveforms were not downsampled.

In order to clarify the differences between target and non-target ERPs at each time point and in each channel, squared point-biserial correlation coefficients (*r*^2^ values) were computed (Blankertz et al., 2011). For each time point and each channel, the mean values of class 2 (target) μ_2_ were subtracted from those of class 1 (non-target) μ_1_, and the values were divided by the standard deviation σ of all samples in order to compute point-biserial correlation coefficient *r* as follows:

(2) $$r:=\frac{\sqrt{N_{2}\cdot N_{1}}}{N_{2}+N_{1}}\frac{\mu_{2}-\mu_{1}}{\sigma },$$

where *N*_2_ and *N*_1_ denote the numbers of data points in class 2 (target) and class 1 (non-target), respectively. The *r*^2^ value, meaning the square of *r*, was then computed. The higher the *r*^2^ value obtained, the larger the difference between the target and non-target is, or the smaller the standard deviation is.

#### Psychological evaluation of affective feelings

In order to measure positive or negative affective feelings, all participants were asked how much they felt each stimulus was positive or negative using a VAS after the experiments. The VAS scores ranged from –100 (most negative) to +100 (most positive), where 0 indicates neutral. PA, permuted-PA, NA, and permuted-NA were played once in pseudo-random order for each participant.

#### Statistical analysis

Differences between classification accuracies and between VAS scores was assessed by means of a two-way repeated-measures ANOVA with factor permutation (the original sound or its permuted sound) and types of affect (positive or negative). We then performed a *t*-test with Bonferroni correction. In order to ensure that each VAS score was positive or negative (not neutral), each VAS score was assessed using a one-sample *t*-test. The classification accuracy for each part was compared under two-permutation conditions by using a paired *t*-test. The point-biserial correlation (Tate, 1954) was evaluated with a test of no correlation, where the *p*-value was corrected using the Bonferroni method. The biserial correlation coefficients were visualized if they were significant.

### Experiment 2: online performance with an ALS patient

#### Participant

A male patient with ALS aged 61 y.o. participated in this study. His ALS Functional Rating Scale-Revised (ALSFRS-R) (Cedarbaum et al., 1999) was zero. This experiment was conducted in his home. He was artificially ventilated via tracheostomy. A transparent letter board for selecting letters with his eyes was used for his daily communication.

#### Online experiment

The online experiment consisted of five training sessions and five test sessions. The participant was asked to rest between sessions. Each session contained two runs, meaning the participant answered two questions per session. In the online experiment, PA, NA, and beep sounds were provided as well as in part A of the previous experiment. The participant was asked to silently count PA stimuli to answer “Yes” and NA stimuli to answer “No.” Each stimulus was provided for 10 sequences, meaning the participant had to count the target sound 10 times per run. A feedback sound was provided in the online experiment.

#### EEG recording

The online BCI system consisted of a laptop computer, digital sound interface, speaker, and EEG amplifier. The instructions and BCI stimuli were presented through the speaker (SoundLink Mini II, Bose Inc., Framingham MA). EEG signals were recorded from C3, Cz, C4, P3, Pz, P4, O1, and O2, according to the “10–20” system. The sampling rate for the EEG recording was 128 Hz. All channels were referenced at Fpz and grounded at AFz. Non-adhesive solid-gel electrodes were used (Toyama et al., 2012). Fixation and visual instruction were not provided so that all the manipulation and instructions of the online BCI were completely independent of gaze control. A hardware bandpass filter (0.1–30 Hz) and notch filter (50 Hz) were applied.

#### EEG analysis

We employed a SWLDA classifier using transfer learning as both online and offline classifiers. The data from 10 healthy participants obtained in experiment 1 (subjects 1–10) was employed to estimate the classifier for the new subject. The analysis window was 1,000 ms. Prior to training the classifier, ERP data that exceed ±100 μV was eliminated from the healthy participant data and the patient training data. Additionally, a Savitzky–Golay filter (3rd order, 61 samples) was applied and the EEG signal was downsampled to 26 Hz. First, the stepwise method was applied to the data obtained from the 10 healthy participants. The training data was not divided into positive and negative, so the training labels were only target and non-target ERPs. Second, the healthy participant data and patient training data were preprocessed using the stepwise method (*p*_in_ = 0.1, *p*_out_ = 0.15). Third, two different classifiers were trained: one was trained on the patient data ***w*** and the other was trained on the healthy participant data ***w***_t_. Then the two classifiers were combined to calculate the score for decision making as follows:

(3) $$\hat{i}={\text{argmax}}_{i}\;\sum_{r=1}^{10}(w\;\cdot \;x_{r,i}+w_{\text{t}}\;\cdot \;x_{r,i}).$$

Binomial testing was applied to the classification accuracy, in order to verify that the achieved accuracy was significantly higher than the chance level (50%). The offline analysis was also performed to identify the required number of sequences.

## Results

### Experiment 1: effect of affective stimuli with able-bodied participants

#### Classification accuracy and VAS

**Figure 2:**
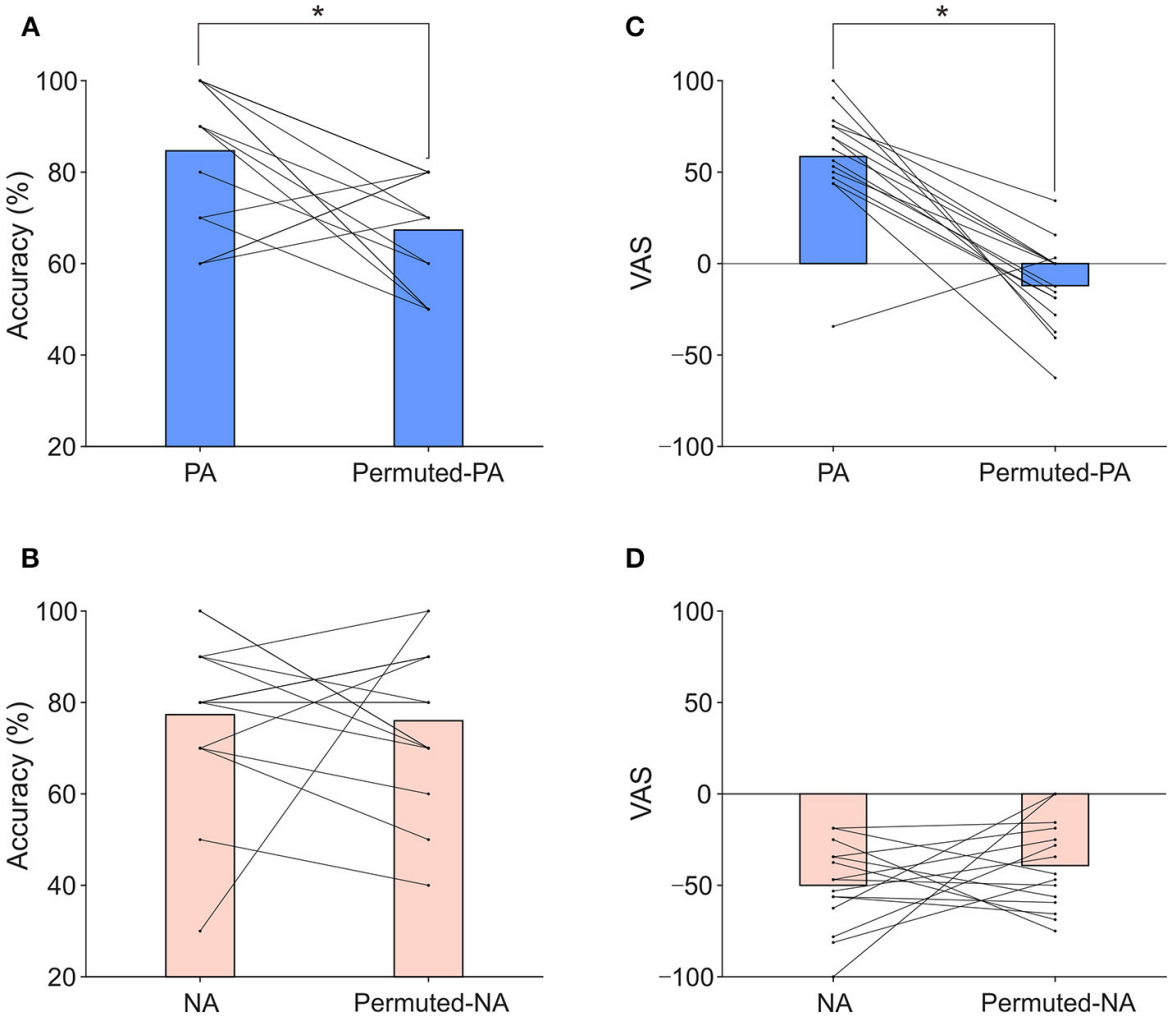
Classification accuracies and VAS scores. **(A)** Classification accuracies of PA and permuted-PA. **(B)** VAS scores of PA and permuted-PA. **(C)** Classification accuracies of NA and permuted-NA. **(D)** VAS scores of NA and permuted-NA. The bar represents the mean classification accuracy or VAS score. The line and dot represent individual classification accuracies or VAS scores. Asterisks indicate significant differences revealed by the *post-hoc* test (*p* < 0.025).

An auditory P300 BCI with positive and negative affective sounds (PA: a meowing cat sound, NA: a screaming cat sound) was tested on 15 healthy participants. Permuted stimuli of the positive and negative affective sounds (permuted-PA, permuted-NA) were also used for comparison. Figure 2A presents the results of classification accuracy when applying PA and permuted-PA sounds. The mean classification accuracies for PA and permuted-PA were 84.7 and 67.3%, respectively. Figure 2B shows the results of classification accuracy when applying NA and permuted-NA sounds. The mean classification accuracies for NA and permuted-NA were 77.3 and 76.0%, respectively. The classification accuracy for each individual subject under each condition is also presented in Table S1. Performing two-way repeated-measures ANOVA on classification accuracy revealed significant main effect on permutation [*F*_(1, 14)_ = 5.55, *p* < 0.05]. There were no significant main effects on types of affect (positive or negative) [*F*_(1, 14)_ = 0.02 *p* = 0.888] and interaction [*F*_(1, 14)_ = 2.90, *p* = 0.111]. The *post-hoc t*-test revealed significant differences for positive sounds [t_(14)_ = 2.94, *p* < 0.025]. No significant difference was found for negative sounds [*t*_(14)_ = 0.21, *p* = 0.838]. The classification accuracies for each part (see Table S2) were not significantly different between permutation conditions [*t*_(14)_ = −1.22, *p* = 0.243].

We used a VAS to measure positive and negative affective feelings for each participant. Figure 2C presents the VAS results when using PA and permuted-PA sounds. The mean VAS scores were 58.5 for PA and −12.1 for permuted-PA. Figure 2D presents the VAS results when applying NA and permuted-NA sounds (individual results are provided in Table S3). The mean VAS scores were −50.0 for NA and −39.2 for permuted-NA, respectively. The VAS score for PA was significantly above zero (*p* < 0.01, one-sample *t*-test) and the VAS score for permuted-PA was not significantly above zero (*p* = 0.072). The VAS scores for NA and permuted-NA were significantly below zero (*p* < 0.01, one-sample *t*-test). Therefore, in terms of VAS scores, PA was positive, permuted-PA was neutral, and NA and permuted-NA were negative. Performing two-way repeated-measures ANOVA on the VAS revealed significant main effect on permutation [*F*_(1, 14)_ = 18.46, *p* < 0.001], types of affect [*F*_(1, 14)_ = 115.47, *p* < 0.001], and their interactions [*F*_(1, 14)_ = 28.18, *p* < 0.001]. The *post-hoc t*-test revealed significant differences for positive sounds [*t*_(14)_ = 6.68, *p* < 0.025]. No significant difference was found for negative sounds [*t*_(14)_ = –1.07, *p* = 0.304].

#### Physiological data

**Figure 3:**
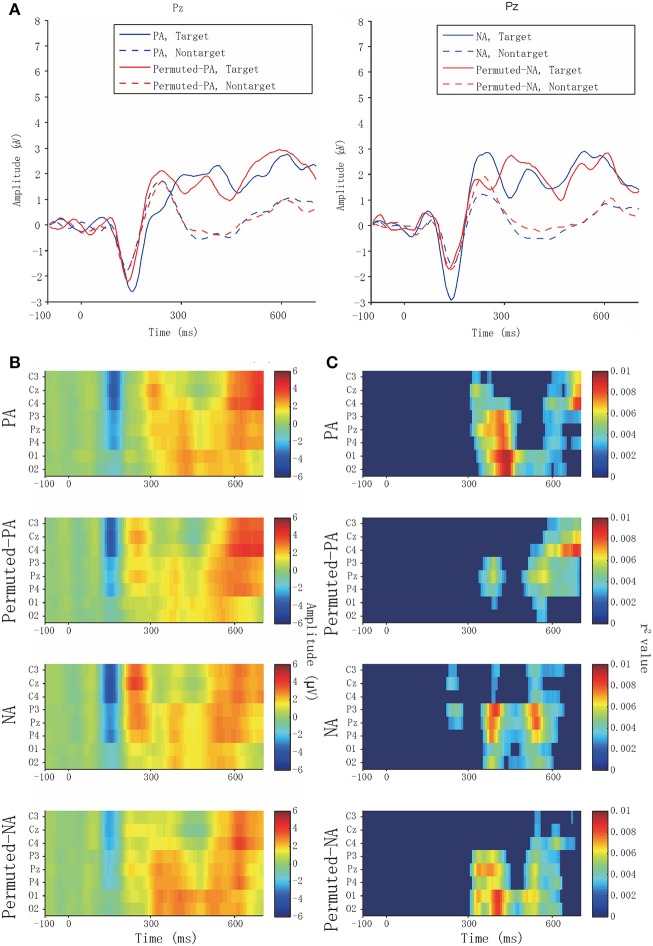
Averaged waveforms and biserial correlation coefficients (*r*^2^ value). **(A)** Target and non-target averaged waveform of PA, permuted-PA, NA, and permuted-NA at Pz. Solid and broken lines represent the target and non-target ERPs, respectively. The blue lines represent PA or NA, and the red lines represent permuted-PA or permuted-NA. **(B)** Averaged waveforms in PA, permuted-PA, NA, and permuted-NA observed from eight channels. **(C)** Biserial correlation coefficients in PA, permuted-PA, NA, and permuted-NA. The biserial correlation coefficients were visualized as zero if the point-biserial correlation was determined to be insignificant through a test of no correlation.

Physiological data analyses were applied to the ERPs. Figure 3A presents the averaged waveforms of target and non-target ERPs observed from Pz. When applying positive affective sounds, the target averaged waveforms of PA in Pz showed peaks at ~400 ms, which can be considered asa late component of the P300. The target waveforms of permuted-PA showed early and late components of the P300 at ~280 and 400 ms, respectively.

Figure 3B presents the averaged waveforms of all channels in PA, permuted-PA, NA, and permuted-NA. The P300 ERP responses, especially the early components, were observed around Cz, and the late components were observed around Pz. Figure 3C presents the biserial correlation coefficient results. The late component of the P300 showed high biserial correlation coefficients for the PA stimulus, suggesting that the late component contributed to the classification. Similar coefficients were also observed with NA and permuted-NA stimuli. However, the early component of the P300 showed no significant correlation.

### Experiment 2: online performance with an ALS patient

**Figure 4:**
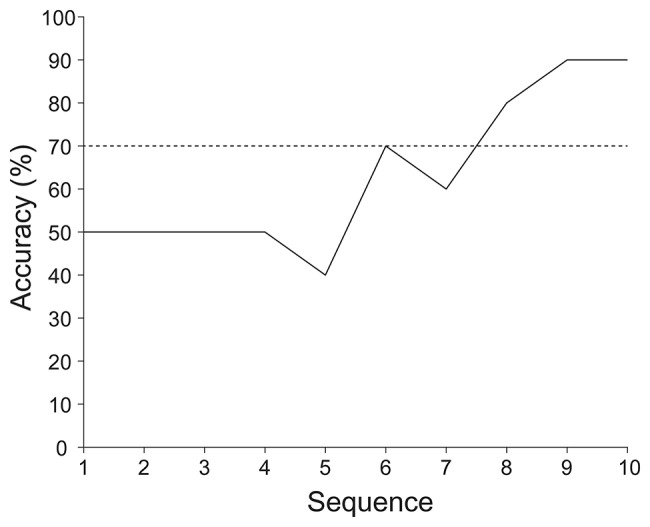
Online and offline classification accuracies achieved by an ALS patient. The online and offline classification accuracies were calculated under same classifier with same data. The online classification accuracy was 90%, which is the same as the offline classification accuracy at sequence 10. Offline classification accuracy was computed by varying the number of sequences from 1 to 10. Also 70% classification accuracy is indicated by the horizontal dotted line.

Figure 4 presents the online and offline classification accuracies of an ALS patient. The online classification accuracy was 90%, which was same as the offline classification accuracy at sequence 10. The online classification accuracy was significantly higher than the chance level (binomial test, *p* < 0.025). According to the offline analysis, six sequences of stimulus presentations were required to achieve over 70% accuracy.

## Discussion

We applied positive and negative affective sounds (PA: a meowing cat sound, NA: a screaming cat sound) with a P300 BCI. Permuted stimuli of the positive and negative affective sounds (permuted-PA, permuted-NA) were also used for comparison. A VAS was used to measure positive and negative affective feelings. We showed that a positive affective stimulus, with accompanying positive affective feelings, improved BCI accuracy. We also demonstrated that the proposed BCI was applicable for an ALS patient.

### Effects of affective stimuli on offline BCI performance

Our study revealed that the PA stimulus improved the offline classification accuracy of an auditory BCI. A previous visual BCI study found that affective facial images improved the classification accuracy of a BCI (Zhao et al., 2011). An fMRI BCI study used click-like tones associated with affective sounds using semantic classical conditioning, and found significant activation in the insula and the inferior frontal triangularis (Van Der Heiden et al., 2014). Our study supports that affective stimuli improve the classification accuracy of an auditory BCI.

We demonstrated the significant differences between the classification accuracies of PA and permuted-PA, and between the VAS scores of PA and permuted PA. In contrast, when applying negative affective sounds, although both NA and permuted-NA showed decreased VAS scores, no significant differences were observed between them in either classification accuracy or VAS. This lack of change between NA and permuted-NA VAS scores may be caused by the scrambling. When scrambling the stimuli, the sounds were cut every 10 ms and the cut sounds were concatenated in pseudo-random order. This procedure removes continuous and frequency features below 100 Hz, but retains frequency features above 100 Hz. The meowing cat sound, used as the PA stimulus, showed decreased VAS scores in the permuted stimulus, suggesting that the features of the cat meowing sound disappeared. The screaming cat sound, used as the NA stimulus, did not show changed VAS scores in the permuted stimulus, suggesting that the features of the screaming cat sound remained. Although past psychological studies using affective stimuli have used two-dimensional evaluations of arousal and valence (Bradley and Lang, 2000; Gerber et al., 2008), the simple one-dimensional VAS used here worked well as a method for psychological measurements.

### Physiological considerations

In order to clarify which components of ERPs contributed to classification, we computed the squared point-biserial correlation coefficients (*r*^2^ values). The peaks of the biserial correlation coefficients were seen at 300–400 ms, which may correspond to the late component of the P300 (Comerchero and Polich, 1999). The averaged waveform analysis indicated that the P300, particularly its late component, contributed to the classification.

This study revealed high biserial correlation coefficients for the late component of the P300 in response to auditory affective stimuli. Modulation of the late component of the P300 has been reported in past studies by using visual or auditory affective stimuli. Cuthbert et al. (2000) provided pictures from the International Affective Picture System to participants in order to evaluate emotional reactions, and reported that highly arousing affective stimuli enhanced the late component of the P300. The valence of visual affective stimuli also enhanced the late component of the P300 (Delplanque et al., 2004). A musical emotion study showed that violations of harmony increased emotions and enhanced the late component of the P300 (Steinbeis et al., 2006). These results indicate that positive affective stimuli modulate the late component of the P300 and contribute to increased classification accuracy with the auditory P300 BCI.

### Online BCI performance

In the online experiment, an ALS patient achieved 90% classification accuracy. Our BCI is a totally vision-free system; all questions, stimuli, and feedback were provided only from the speaker. Thus, this system may be worth applying to patients who cannot see. The arbitrary yes/no questions can be provided orally by replacing the questions provided from the speaker. Our BCI system can only ask yes/no binary questions, but the affective sounds may be applied to auditory multiple-choice BCIs. Auditory multiple-choice BCIs have previously been proposed and evaluated. Halder et al. (2016a) evaluated a 25-command auditory BCI system with and without a visual support matrix, and two subjects achieved 92% classification accuracy online after training. Halder et al. (2016b) also proposed an auditory BCI that enables one to spell the Japanese Hiragana syllabary (50 commands), and four out of six healthy subjects achieved over 70% classification accuracy. A patient with the spinal cord injury also controlled the BCI and achieved 56% classification accuracy after training. The effects of affective stimuli on multi-command auditory BCI systems may be better evaluated in future studies.

### Limitations and future perspectives

This study demonstrated that positive affective stimuli improve classification accuracy. However, further studies are required to determine if affective stimuli generally improve BCI classification accuracy. Moreover, classification accuracy for each part did not show the significant difference between part A and part B. The result may be caused by the increased variance of the two class data since responses obtained by positive and negative stimuli were combined. The effect of affect in part B may also influenced because permuted-NA was negative. Additionally, we applied transfer learning in the online system, but the effects of the algorithm should be evaluated and the parameters should be optimized in future studies. The affective stimuli evaluated in this study may also be applied to a multi-command BCI as a next step.

## Conclusion

In conclusion, we demonstrated that a positive affective stimulus, accompanied by positive affective feelings, improved BCI accuracy. These results suggest that affective stimuli may be useful in developing a practical auditory BCI system for patients with physical disabilities.

## Author contributions

AO, HO, and KK designed the experiment. AO and KT collected the data. AO, HO, and TK analyzed the data. AO, KT, TK, and KK wrote the manuscript.

### Conflict of interest statement

The authors declare that the research was conducted in the absence of any commercial or financial relationships that could be construed as a potential conflict of interest.
