# Supplementary material for: Affective Stimuli for an Auditory P300 Brain-Computer Interface
Source: Front Neurosci. 2017 Sep 21;11:522. doi: 10.3389/fnins.2017.00522 (PMC5613193; doi:10.3389/fnins.2017.00522)
Supplement: Supplementary file 1 [file Table1.DOCX]

**Supplemental Materials**

Table S1. Classification accuracy for PA, NA, Permuted-PA and Permuted NA

| condition | Subject | | | | | | | | | | | | | | | Mean |
| --- | --- | --- | --- | --- | --- | --- | --- | --- | --- | --- | --- | --- | --- | --- | --- | --- |
|  | 1 | 2 | 3 | 4 | 5 | 6 | 7 | 8 | 9 | 10 | 11 | 12 | 13 | 14 | 15 |  |
| PA | 80 | 60 | 70 | 100 | 70 | 60 | 100 | 100 | 90 | 100 | 90 | 90 | 60 | 100 | 100 | 84.7 |
| Permuted-PA | 60 | 80 | 80 | 50 | 50 | 70 | 80 | 80 | 50 | 80 | 70 | 60 | 80 | 50 | 70 | 67.3 |
| NA | 100 | 70 | 70 | 80 | 80 | 90 | 80 | 50 | 80 | 90 | 80 | 70 | 100 | 90 | 30 | 77.3 |
| Permuted-NA | 70 | 50 | 90 | 90 | 70 | 100 | 90 | 40 | 80 | 80 | 80 | 60 | 70 | 70 | 100 | 76.0 |
