# Supplementary material for: Affective Stimuli for an Auditory P300 Brain-Computer Interface
Source: Front Neurosci. 2017 Sep 21;11:522. doi: 10.3389/fnins.2017.00522 (PMC5613193; doi:10.3389/fnins.2017.00522)
Supplement: Supplementary file 2 [file Table2.DOCX]

**Supplemental Materials**

Table S2. Classification accuracy (%) for each part

| condition | Subject | | | | | | | | | | | | | | | Mean |
| --- | --- | --- | --- | --- | --- | --- | --- | --- | --- | --- | --- | --- | --- | --- | --- | --- |
|  | 1 | 2 | 3 | 4 | 5 | 6 | 7 | 8 | 9 | 10 | 11 | 12 | 13 | 14 | 15 |  |
| Part A | 85 | 65 | 60 | 55 | 45 | 70 | 85 | 65 | 55 | 90 | 50 | 65 | 70 | 80 | 55 | 66.3 |
| Part B (permuted sounds) | 65 | 60 | 75 | 75 | 60 | 80 | 70 | 65 | 50 | 90 | 90 | 75 | 80 | 60 | 80 | 71.7 |
