# Supplementary material for: Affective Stimuli for an Auditory P300 Brain-Computer Interface
Source: Front Neurosci. 2017 Sep 21;11:522. doi: 10.3389/fnins.2017.00522 (PMC5613193; doi:10.3389/fnins.2017.00522)
Supplement: Supplementary file 3 [file Table3.DOCX]

**Supplemental Materials**

Table S3. VAS for PA, NA, Permuted-PA and Permuted NA

| condition | Subject | | | | | | | | | | | | | | | Mean |
| --- | --- | --- | --- | --- | --- | --- | --- | --- | --- | --- | --- | --- | --- | --- | --- | --- |
|  | 1 | 2 | 3 | 4 | 5 | 6 | 7 | 8 | 9 | 10 | 11 | 12 | 13 | 14 | 15 |  |
| PA | 62.5 | 46.88 | -34.4 | 56.25 | 75 | 43.75 | 68.75 | 75 | 90.63 | 68.75 | 100 | 78.13 | 53.13 | 43.75 | 50 | 58.5 |
| Permuted-PA | 0 | -18.8 | 3.125 | -15.6 | 34.38 | -18.8 | 0 | 0 | -37.5 | -28.1 | -40.6 | 15.63 | -12.5 | -62.5 | 0 | -12.1 |
| NA | -18.8 | -34.4 | -53.1 | -18.8 | -56.3 | -46.9 | -34.4 | -46.9 | -25 | -37.5 | -100 | -56.3 | -81.3 | -78.1 | -62.5 | -50.0 |
| Permuted-NA | -43.8 | -18.8 | -34.4 | -15.6 | -65.6 | -50 | -56.3 | -25 | -75 | -68.8 | 0 | -59.4 | -46.9 | -28.1 | 0 | -39.2 |
